# Altered CD38/Cyclic ADP-Ribose Signaling Contributes to the Asthmatic Phenotype

**DOI:** 10.1155/2012/289468

**Published:** 2012-11-20

**Authors:** Joseph A. Jude, Mythili Dileepan, Reynold A. Panettieri, Timothy F. Walseth, Mathur S. Kannan

**Affiliations:** ^1^Department of Veterinary and Biomedical Sciences, University of Minnesota, Saint Paul, MN 55108, USA; ^2^Department of Medicine, University of Pennsylvania, Philadelphia, PA 19104, USA; ^3^Department of Pharmacology, University of Minnesota, Minneapolis, MN 55455, USA

## Abstract

CD38 is a transmembrane glycoprotein expressed in airway smooth muscle cells. The enzymatic activity of CD38 generates cyclic ADP-ribose from **β**-NAD. Cyclic ADP-ribose mobilizes intracellular calcium during activation of airway smooth muscle cells by G-protein-coupled receptors through activation of ryanodine receptor channels in the sarcoplasmic reticulum. Inflammatory cytokines that are implicated in asthma upregulate CD38 expression and increase the calcium responses to contractile agonists in airway smooth muscle cells. The augmented intracellular calcium responses following cytokine exposure of airway smooth muscle cells are inhibited by an antagonist of cyclic ADP-ribose. Airway smooth muscle cells from CD38 knockout mice exhibit attenuated intracellular calcium responses to agonists, and these mice have reduced airway response to inhaled methacholine. CD38 also contributes to airway hyperresponsiveness as shown in mouse models of allergen or cytokine-induced inflammatory airway disease. In airway smooth muscle cells obtained from asthmatics, the cytokine-induced CD38 expression is significantly enhanced compared to expression in cells from nonasthmatics. This differential induction of CD38 expression in asthmatic airway smooth muscle cells stems from increased activation of MAP kinases and transcription through NF-**κ**B, and altered post-transcriptional regulation through microRNAs. We propose that increased capacity for CD38 signaling in airway smooth muscle in asthma contributes to airway hyperresponsiveness.

## 1. Introduction

Contractility of airway smooth muscle (ASM) depends on the dynamic regulation of intracellular calcium concentration [1]. Contractile agonists act on G-protein-coupled receptors to cause oscillatory changes in [Ca^2+^]_*i*_, mediated by influx of calcium from the extracellular space and release of calcium from the intracellular stores [2–5]. The major [Ca^2+^]_*i*_ store in ASM cells is the sarcoplasmic reticulum (SR). Release of calcium from the SR is brought forth principally by two signaling molecules, inositol 1,4,5-trisphosphate (IP_3_) and cyclic ADP-ribose (cADPR). Cyclic ADP-ribose is derived from *β*-NAD through the enzymatic activity of ADP-ribosyl cyclase which is a constituent of the cell-surface protein CD38 [6] (reviewed in [2]). Calcium influx into the cell can occur through voltage- and receptor-operated calcium channels in the plasma membrane [7, 8]. It can also occur by influx that is triggered by depletion of SR calcium stores through a mechanism known as store-operated calcium entry [4]. Transient receptor potential proteins are thought to mediate influx of calcium through receptor- and store-operated channels [9]. 

 In ASM cells, contractile agonists cause a biphasic elevation of [Ca^2+^]_*i*_ that is characterized by an initial rapid rise, followed by a decline to a plateau concentration above the basal level. The initial rapid phase of the biphasic elevation of [Ca^2+^]_*i*_ has been attributed to SR calcium release, while the sustained phase of elevation is to influx from extracellular space. However, with the use of improved temporal and spatial resolution features of real-time confocal microscopy, our understanding of agonist elicited [Ca^2+^]_*i*_ elevation has significantly changed. These studies have demonstrated that the biphasic elevation of the [Ca^2+^]_*i*_ consists of propagating regenerative calcium oscillations of similar amplitude within a given region of the ASM cell. The frequency and the propagation velocity of these calcium oscillations increase with increasing concentration of the agonist. The biphasic nature of the [Ca^2+^]_*i*_ response is in fact the spatiotemporal integration of oscillations in calcium across the entire cell. While the initiation of the calcium oscillations depends on SR calcium release, their sustenance requires repletion of the SR stores through influx from the extracellular space. Furthermore, activation of the IP_3_ receptors in the SR is crucial for the initiation of calcium oscillations by agonists, while maintained calcium oscillations require calcium release through the ryanodine receptor channels in the SR. Calcium release through the ryanodine receptor channels in the SR in ASM cells is mediated by cADPR. Recent studies have also demonstrated that inflammatory cytokines such as TNF-*α*, IL-1*β*, and IFN-*γ* and the Th2 cytokine IL-13 regulate the expression and function of the pathways that govern [Ca^2+^]_*i*_ responses to agonists, thereby contributing to ASM hyperresponsiveness [10, 11]. One of the pathways in [Ca^2+^]_*i*_ regulation in ASM cells involves CD38, and the expression of CD38 in ASM cells derived from asthmatics is upregulated by TNF-*α* to a significantly greater extent than in cells from nonasthmatics [12]. The regulation of CD38 expression and the role of different transcription factors, signaling intermediates and microRNAs in this regulation, have been the focus of investigations in our laboratory. 

CD38 is a 45-kDa type II glycoprotein expressed in a variety of cells from a diverse array of organisms. It belongs to a family of nucleotide metabolizing enzymes capable of generating cyclic adenosine diphosphoribose (cADPR) and ADPR from *β*-NAD or NAADP from NADP [13]. cADPR, ADPR, and NAADP have been shown to be involved in intracellular calcium regulation in both immune cells and excitable cells such as smooth muscle cells. Earlier studies from our laboratory showed evidence for intracellular calcium release by cADPR during activation of G_*αq*_ and G_*αi*_-type G-protein-coupled receptors in airway smooth muscle cells [14]. Studies in other types of cells indicate that this calcium release involves the dissociation of FKBP-12.6 and activation of ryanodine receptors in the sarcoplasmic reticulum [15, 16]. Evidence from FKBP-12.6 knockout mice revealed that cADPR may be an endogenous ligand for this protein and binding of cADPR is an essential step in the activation of ryanodine receptor calcium release channels [17]. In airway smooth muscle cells as well as cardiac myocytes, the calcium-induced calcium release mechanism may be mediated through activation of ryanodine receptor channels by cADPR. The evidence that cADPR is involved in calcium release in airway smooth muscle stems from the following observations: direct addition of cADPR to the cytosolic compartment of airway smooth muscle cells releases calcium from ryanodine receptor channels [18]; cADPR antagonists inhibit intracellular calcium release brought forth by contractile agonists [14]; increasing CD38 expression by inflammatory cytokines (i.e., TNF-*α*, IL-1*β*, IL-13) and thereby augmenting CD38/cADPR signaling cause significant enhancement of calcium release by agonists that is sensitive to inhibition by cADPR antagonists [10]. These results provide evidence for CD38/cADPR signaling in the regulation of intracellular calcium and its potential for enhanced contribution to such regulation during inflammation in airway smooth muscle.

Investigations from other laboratories show that in chemokine-stimulated neutrophils and dendritic cells, ADPR may activate plasma membrane-associated TRPM2 calcium channels and thereby regulate neutrophil and dendritic cell chemotaxis [19]. Evidence from Lund's laboratory has revealed that chemotaxis and bacterial clearance are significantly compromised in neutrophils obtained from CD38 deficient mice [20]. Furthermore, evidence also demonstrates that in CD38 deficient mice there is significant attenuation of T-cell dependent humoral immune responses following immunization with antigens [21]. This defect in humoral immune response appears to result from lack of migration of dendritic cells from inflammatory sites to regional lymph nodes and insufficient dendritic cell priming of CD4 T cells at these sites [20]. These results provide evidence for a role of CD38 in both innate and adaptive immune responses of the host. 

## 2. CD38/cADPR Signaling and Airway Smooth Muscle Function 

 In an attempt to elucidate the contribution of this signaling pathway to airway function, we developed two different models systems to evaluate intracellular calcium responses to spasmogens: ASM cells obtained from CD38 knockout and wild-type mice; human ASM cells expressing a smooth muscle phenotype and maintained in short-term cultures. The intracellular calcium responses to acetylcholine and endothelin-1 of cells obtained from CD38KO mice were significantly lower than responses in cells from WT mice [22]. The calcium responses in the myocytes from CD38KO mice were also insensitive to modulation by the cADPR antagonist, indicating that the defect in calcium signaling can be attributed to lack of cADPR generation [22]. This defective calcium signaling in airway smooth muscle cells is reflected in significant attenuation of methacholine-induced airway resistance and dynamic compliance measured in intact CD38KO mice, suggesting an airway phenotype of these mice [22].

 In HASM cells in culture, the intracellular calcium responses to multiple spasmogens were found to be significantly augmented upon treatment with inflammatory cytokines that are implicated in asthma [10]. The augmented calcium responses were attributable to increased CD38/cADPR signaling since they were reduced by a cADPR antagonist as well as by antisense downregulation of CD38 expression [10, 23]. The fact that inflammatory cytokines such as TNF-*α* and IL-13, a Th2 cytokine, are capable of increasing the capacity for CD38/cADPR signaling in human ASM cells indicates its potential role in human asthma. 

 Based on the results reported above in the CD38KO mice and in HASM cells following exposure to inflammatory cytokines, we hypothesized that CD38/cADPR signaling will be enhanced during airway inflammation, thus contributing to airway hyperresponsiveness, a hallmark feature of asthma. In order to address this hypothesis specifically, we used two different model systems: (i) mouse models of inflammatory airway disease to assess the contribution of CD38 to airway inflammation and AHR; (ii) CD38 expression, function, and its regulation in ASM cells obtained from asthmatic and nonasthmatic donors.

## 3. Contribution of CD38 to the Asthmatic Phenotype

CD38 deficient mice were generated by Cockayne et al. to study the role of this molecule in host immune responses against pathogens [21]. CD38^−/−^ mice showed an immunological phenotype characterized by attenuated chemotaxis and antigen presentation by dendritic cells [20]. In our laboratory, we evaluated the respiratory phenotype of CD38^−/−^ mouse following induction of airway inflammation by murine recombinant IL-13 or TNF-*α*. The inflammatory cytokine TNF-*α* and the Th2 cytokine IL-13 play important roles in the development of allergic asthma. In light of the findings by Partida-Sánchez et al. that CD38-null mice show suboptimal inflammatory response [20], we chose the model of cytokine-induced airway inflammation to circumvent the possibility of reduced inflammatory response to allergen challenge. Following brief and repeated exposure to IL-13 or TNF-*α*, comparable airway inflammation was induced in wild type and CD38^−/−^ mice [24, 25]. However, the airway resistance in response to the contractile agonist methacholine was significantly attenuated in the CD38^−/−^ mice compared to the wild type mice [24, 25]. In light of our findings that CD38 deficient mouse airway myocytes show reduced Ca^2+^ responses, we hypothesized that the differential methacholine responsiveness of CD38^−/−^ mice can be attributed to the altered ASM contractility. Tracheal rings isolated from the WT and CD38^−/−^ were used for measurement of isometric contractile responses following exposure to IL-13 or TNF-*α* *in vitro*. CD38-deficient tracheal rings generated significantly reduced isometric force in response to agonist compared to the tracheal rings from WT mice [24, 25]. These observations support our hypothesis that CD38 contributes to the development of airway hyperresponsiveness (AHR) through its pivotal role in ASM Ca^2+^ dynamics and contractility. 

 Based on the findings reported by Lund and her coworkers, the immunological functions of CD38 in the development of AHR cannot be understated. Studies by Lund showed that CD38 expression in immune cells is critical for the inflammatory and immunological steps that culminate in allergy and AHR (reviewed in [26]). The critical events in host immune response to allergens, such as antigen presentation by dendritic cells, were negatively impacted in CD38-deficient mice [20]. Studies using ovalbumin-induced allergic inflammation in mouse revealed that along with attenuated methacholine responsiveness, CD38-deficient mice also developed reduced airway inflammation, signified by substantially reduced eosinophilia, Th2 cytokines, and allergen-specific immunoglobulins E (IgE) and G1 (IgG1) levels compared to the WT mice [27]. These findings support a pivotal role for CD38 in the pathogenesis of asthma through its dual functions in the immune response to allergen and ASM contractility. The involvement of CD38/cADPR signaling pathway in the pathogenesis of asthma at multiple levels of the process is supported by these observations. 

## 4. Asthmatic Phenotype of Airway Smooth Muscle

**Figure 1:**
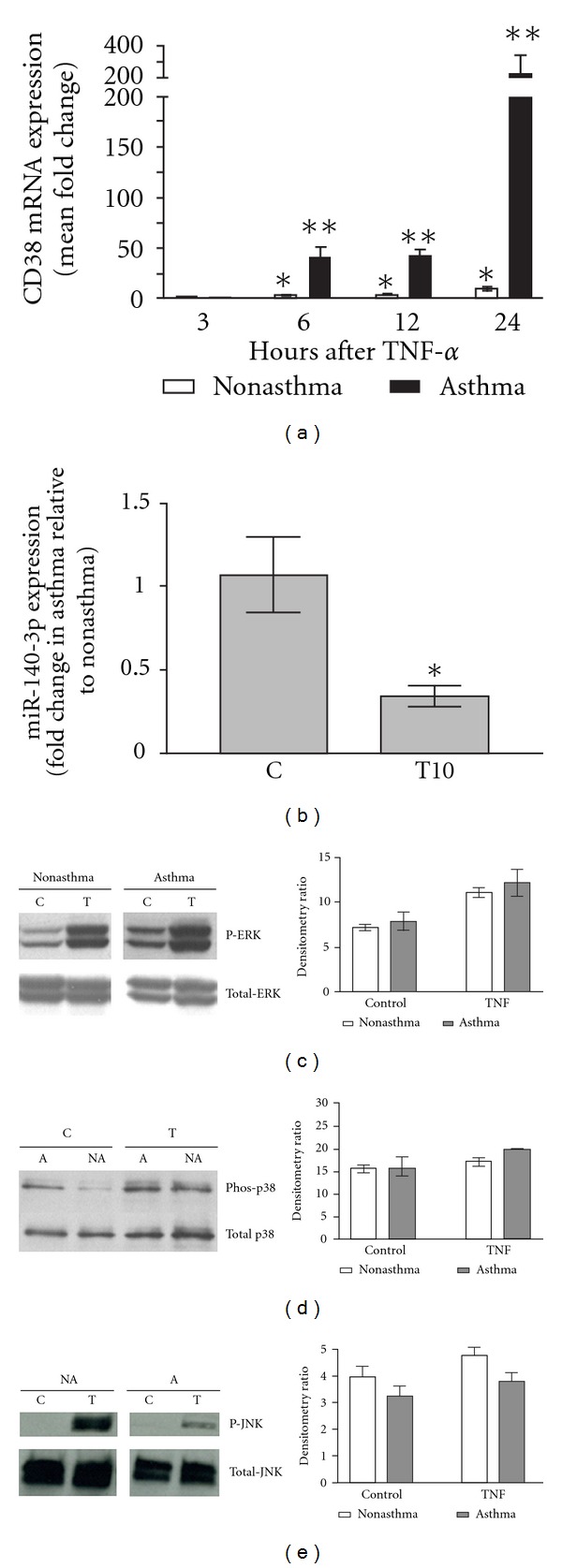
Altered signaling mechanisms contributing to asthmatic airway smooth muscle cell phenotype. Studies in our laboratory revealed some important changes in the HASM cells from asthmatic donors compared to the cells from nonasthmatic donors, suggesting a potential role for these mechanisms in the asthmatic phenotype of ASM cells. (a) Inflammatory cytokine TNF-*α* (10 ng/mL) induced differentially elevated CD38 expression in asthmatic HASM cells compared to the nonasthmatic HASM cells, as early as 6 hrs following addition of the cytokine (*n* = 3/nonasthmatic or asthmatic HASM cells, *P < 0.05, significantly different from the vehicle-treated controls; **P < 0.05, significantly different from the nonasthmatic HASM cells treated with TNF-*α*). (b) In HASM cells from asthmatic donors, TNF-*α* (10 ng/mL, 24 hrs of exposure) caused significant attenuation of miR-140-3p expression compared to the nonasthmatic HASM cells. The basal miR-140-3p expression levels were comparable between nonasthmatic and asthmatic HASM cells (*n* = 5/nonasthmatic group; *n* = 6/asthmatic group, *P < 0.05, significantly different from the nonasthmatic group). (c–e) (Left and right panels) HASM cells obtained from asthmatic donors showed elevated basal and TNF-*α*-induced activations of ERK and p38 MAP kinases compared to the nonasthmatic HASM cells. TNF-*α*-induced JNK MAP Kinase activation was attenuated in asthmatic HASM cells compared to the nonasthmatic cells (blots representative of 5 independent experiments). (a) and (c–e) are reproduced with permission from [12]. Panel (b) is Reproduced with permission from [28]. Altered signaling mechanisms contributing to asthmatic airway smooth muscle cell phenotype.

 The intracellular calcium responses to contractile agonists, isometric contractile responses of tracheal rings, and responsiveness of airways to inhaled methacholine are significantly attenuated in airway smooth muscle obtained from CD38^−/−^ mice. In HASM cells exposed to inflammatory cytokines, the CD38/cADPR signaling pathway contributes to enhanced calcium signaling in response to activation of G-protein-coupled receptors [10]. The airway hyperresponsiveness following cytokine challenge or allergen sensitization and challenge is significantly compromised in CD38^−/−^ mice [10]. These observations led us to speculate that ASM cells derived from asthmatic donors will have increased basal and cytokine-induced expression of CD38. To address this hypothesis, we obtained ASM cells from nonasthmatic and asthmatic donors. The cells were maintained in culture for up to 5 passages. Cells were exposed to TNF-*α* or vehicle following growth arrest and CD38 expression, and their enzymatic activities were measured. There was very little, if any, basal expression of CD38 in cells from either asthmatic or nonasthmatic donors [12]. However, exposure to a range of TNF-*α* concentrations caused a significantly greater induction of CD38 expression in ASM cells from asthmatics than in cells from nonasthmatics [12]. This differential induction of CD38 expression occurred as early as 6 hrs following exposure to TNF-*α* and maintained for over 24 hrs and was unrelated to level of TNFR1 expression in the cells (Figure 1) [12]. This pattern of differential induction of CD38 expression was seen in cells obtained from donors with a history of clinical asthma as well as from fatal asthmatics. This asthmatic phenotype in terms of differential CD38 induction was maintained for up to 5 passages in culture.

 We examined some potential mechanisms involved in the differential induction of CD38 expression and for the asthmatic phenotype of ASM cells. Our previous studies showed that members of the MAP kinases and the transcription factors NF-*κ*B and AP-1 were required for TNF-*α* induction of CD38 expression in HASM cells [29]. Therefore, we hypothesized that the increased CD38 expression will be reflected by greater activation of MAP kinases and the transcription factors NF-*κ*B and AP-1. It is worth noting that the *CD38* promoter has response elements for these transcription factors and mutagenesis of either of these transcription factor binding sites would result in lack of promoter activation by TNF-*α* [30]. In HASM cells derived from asthmatic donors, we found consistently elevated p38 and ERK activation (phosphorylated forms), but not that of JNK as compared to cells from nonasthmatic donors (Figure 1) [12]. Activation of NF-*κ*B and AP-1 was measured by analyzing their nuclear content as well as their binding to consensus sequences. Both these parameters were comparable in cells from asthmatics and nonasthmatics [12]. These results support the concept that asthmatic ASM cells are intrinsically programmed to express greater levels of MAP kinase activation and the greater induction of CD38 expression by TNF-*α* may involve increased rate of transcription rather than transcript stability. This conclusion is further supported by the fact that transcript stability was comparable in cells from asthmatics and nonasthmatics [12]. However, we cannot rule out the contribution of other transcription factors to the observed differential response of asthmatic ASM cells. Furthermore, signaling mechanisms independent of MAP kinases may also be involved in this differential induction. It should be noted that significant ERK and p38 phosphorylation were reported in both epithelial cells and smooth muscle cells obtained from severe asthmatics, suggesting that these MAP kinase pathways may have an important role in the pathogenesis of severe asthma [31–33]. The levels of expression of kinases upstream of ERK as well as the MAP kinase phosphatase-1 are comparable in cells from asthmatics and nonasthmatics [12]. Precisely how a higher level of activation of these MAP kinases is maintained in the asthmatic ASM cells is not entirely clear. 

 With respect to other signaling pathways involved in the regulation of CD38 and their potential contribution to the asthmatic phenotype, we explored the role of PI3 kinases in such regulation. We found that ASM cells from both asthmatics and nonasthmatics express comparable levels of class I PI3 kinase P110 isoforms *α*, *β*, and *δ* [34]. Inhibition of the *α* and *δ* isoforms by siRNA transfection causes comparable magnitude of inhibition of TNF-*α*-induced CD38 expression in cells from asthmatics and nonasthmatics [34]. However, under comparable levels of siRNA-mediated downregulation of expression, the residual CD38 expression in asthmatic ASM cells was significantly higher than expression in cells from nonasthmatics [34]. Inhibition of the *β* isoform has no effect on CD38 expression. These results, while demonstrating similar expression levels of class I PI3 kinase isoforms in asthmatic and nonasthmatic ASM cells, show decreased sensitivity of CD38 expression in asthmatic ASM cells to inhibition of PI3 kinases. This is yet another example of an asthmatic phenotype of airway smooth muscle cells in terms of differential contribution of signaling pathways to the regulation of expression of specific genes. In this context, studies have shown that PI3 kinase signaling contributes to the proliferative response of ASM cells from asthmatics, while the ERK MAP kinase pathway seems to be involved in cells from nonasthmatic donors [32]. 

## 5. Posttranscriptional Regulation 

Posttranscriptional regulation of genes is a mechanism aimed at altering the target gene expression swiftly in response to an environmental cue. In light of recent advancements in understanding microRNA regulation of gene expression, it appears that posttranscriptional regulation is also a fine tuning mechanism to delicately balance gene expression in cells (reviewed in [35]). Studies in our laboratory have shown that CD38 expression is regulated posttranscriptionally, at the level of transcript stability [29]. MAP kinases p38 and ERK1/2 regulate CD38 expression by modulating the transcript stability. The exact mechanism involved in the regulation of CD38 mRNA stability is not known. However, our studies focused on both RNA-binding proteins and microRNA as the potential mechanisms involved in the post-transcriptional regulation of CD38. 

Specific short sequence motifs, primarily located on the 3′ untranslated region (3′UTR), interact with RNA-binding proteins to modulate the stability or translatability of the transcript [36, 37]. Adenylate-uridine- (AU-) rich motifs and cytosine-guanosine-uridine- (CGU-) rich motifs are the major sequence motifs found in 3′UTR of many transcripts and act as the *cis* elements responsible for mediating mRNA stability [38, 39]. The CD38 3′UTR possesses 4 AU-rich motifs, indicating a potential role for these *cis* elements in the post-transcriptional regulation of CD38 expression*. In vitro* pull down assays, using synthetic RNA oligonucleotide containing one of the AU-rich elements of CD38 3′UTR and HASM cell lysates, showed that the RNA-stabilizing protein, human protein R (HuR), the translational modulator protein and T-cells intracellular antigen-1 (TIA-1) bind selectively to the AU-rich element, in the presence of TNF-*α*. However, transient over expression of HuR in the HASM cells did not result in altered CD38 mRNA expression, suggesting RNA destabilizing proteins, such as tristetraprolin (TTP), may have a counteracting role in TNF-*α*-induced CD38 expression in HASM cells (unpublished results). 

MicroRNAs (miRNA) are emerging as molecules with important roles in gene regulation in different orders of living organisms. Encoded from intronic or intergenic regions of the genome, miRNAs regulate the expression of a significant portion of human genes [40]. The general mechanism of regulation by miRNA is through modulating the stability or translatability of the target mRNA [35]. On average, a single miRNA can target and regulate ~100 different genes, although it is suggested that the cluster of genes regulated by a single miRNA would belong to a particular cellular function. Bioinformatic screening of CD38 3′UTR revealed predicted targets for several miRNAs. Systematic studies on the expression and role of some of these miRNAs have been carried out in our laboratory. Luciferase-CD38 3′UTR reporter assays confirmed that miR-140-3p, one of the miRNAs predicted to target CD38, functionally targets CD38 3′UTR [28]. Further studies using over expression of miR-140-3p mimic in HASM cells showed that CD38 mRNA and protein expressions were significantly downregulated by miR-140-3p [28]. Findings of our study suggest that the inhibitory effect of miR-140-3p mimic on CD38 expression is only partially mediated through direct binding to the CD38 3′UTR, since there was only a marginal inhibition of luciferase activity by miR-140-3p mimic. The dominant effect of miR-140-3p on CD38 expression appears to be mediated indirectly through transcriptional mechanisms, by inhibiting activation of p38 MAP Kinase and NF-*κ*B. MicroRNA target prediction algorithms revealed an array of genes potentially targeted by miR-140-3p in humans (Table 1). We also found that miR-140-3p expression was significantly attenuated by TNF-*α* exposure in asthmatic HASM cells compared to nonasthmatic HASM cells [28]. Whether the greater degree of downregulation of miR-140-3p in asthmatic ASM cells compared to ASM cells from nonasthmatics contributes to the reported differential induction of CD38 expression in these cells is not clear, but remains an attractive hypothesis. These findings also suggest that CD38 and miR-140-3p may be part of a differential gene expression profile in asthmatic HASM cells, indicating a potential role for these two molecules in the pathogenesis of asthma. Recent investigations have provided insights into the role of microRNAs in the regulation of ASM cell phenotype and ASM contractility [41, 42]. Studies have also reported altered microRNA expression profiles in various cells obtained from asthmatic donors [43, 44]. The roles of specific microRNAs in the development of AHR have also been demonstrated by studies in mouse models of asthma [45–47]. MicroRNA target prediction algorithms revealed an array of genes potentially targeted by miR-140-3p in humans (Table 1). The table has a partial list of the genes that encode proteins associated with G-protein-coupled receptor function, ion channels, chemokines, transcription factors, signaling proteins, contractile and cytoskeletal elements, and cell proliferation. It should be noted that some of the genes targeted by the microRNA are involved in the regulation of CD38 expression, that is, transcription factors, MAP kinases, and PKC signaling-associated proteins. Downregulation of expression of the chemokines by miR-140-3p has the potential to have significant anti-inflammatory effects.

## 6. Conclusions

 Our investigations of CD38 expression have revealed some important differences between ASM cells from asthmatics and nonasthmatics in the sensitivity to inflammatory cytokines in terms of level of expression, sensitivity of expression to inhibition of signaling intermediates such as MAP Kinases and PI3 Kinases, in microRNA expression, and post-transcriptional regulation of expression. These differences in the asthmatic ASM cells are maintained over at least 5 passages in culture. Studies in cytokine or allergen-induced inflammatory airway disease mouse models have revealed that CD38 contributes to airway hyperresponsiveness both through its role in generating calcium-mobilizing second messenger molecules and thereby the contractility of airway smooth muscle and through its role in adaptive immune response. We propose that increased capacity for CD38/cADPR signaling in airway smooth muscle in asthma contributes to the development of airway hyperresponsiveness. Whether the differences that we report transcend all asthma phenotypes is not currently known and requires further investigation.

## Figures and Tables

**Table 1:** A partial list of miR-140-3p gene targets predicted by TargetScan and microRNA.org.

Receptors and channels	ARHGAP3, IRGQ, SCN3A, NKIRAS2, GPR12, GIT1, GABRB2, PLEKHA1, KCNA7, TRPM7, SGSM1, GPR158, REEP5, SGIP1, GAB2, RASGEF1B, FKBP1A, and RGS1
Cytokine/chemokine regulation	SOCS4, CDK6, MMP16, TGIF2, ADAM17, ITK, CHL1, ADAM9, CXCL11, IL8, IL6, and CXCL6
Related to transcriptional regulation	CREB1, SP3, TAF2, SP4, TCEB3, SIRT1, E2F7, KLF4, RAB2A, FOX2, NFYA, NKRF, HDAC4, GABPB1, SP3, KLF5, IKB, and KB
MAPK and PKC/MTOR pathways	PTEN, RAP1B, RPS6KA3, RICTOR, MAPK1, PPFIBP1, CDS2, MARCKS, MAP2K6, and IL24,
Related to actin and myosin	MYLK4, MAPRE3
Related to proliferation	ZNF3
